# Seeds of Scholarship: A Systematic Review of Research Engagement in Undergraduate Medical Education

**DOI:** 10.5334/pme.2199

**Published:** 2026-01-21

**Authors:** Luksanaporn Krungkraipetch, Kitti Krungkraipetch, Kaneungnit Usimat, Somying Bookaew

**Affiliations:** 1 Faculty of Medicine, Burapha University, Chonburi, Thailand; 2 Research Department, Faculty of Medicine, Burapha University, Chonburi, Thailand

## Abstract

**Introduction:**

Research engagement during undergraduate medical education is increasingly recognized as essential for developing physician-scientists and fostering evidence-based practice. However, evidence regarding its effectiveness, challenges, and long-term impact remains fragmented. This systematic review synthesizes current evidence on outcomes, motivational factors, barriers, and effective practices in undergraduate medical research programs.

**Methods:**

Following PRISMA 2020 guidelines, we searched PubMed/MEDLINE, ERIC, Scopus, and Google Scholar for studies on undergraduate medical research engagement. Two reviewers screened 12,600 records and assessed 262 full texts for eligibility and quality (MERSQI, CASP). Eleven studies (n = 5,564 students, nine countries, 2010–2024) met the inclusion criteria. Owing to heterogeneity, a narrative synthesis was conducted across thematic domains.

**Results:**

Publication rates ranged from 15–55% (I^2^ = 87%), correlating with institutional resources. Students publishing before graduation were 1.9 times more likely to publish afterward (95% CI 1.6–2.3, p < 0.001). Experiential activities enhanced motivation (β = 0.45, p < 0.001) and self-efficacy (β = 0.38, p < 0.001), while grades showed no effect. Major barriers included curriculum demands (78.3%), limited funding (71.5%), and time constraints (61.7%). Prior academic performance did not predict research motivation.

**Discussion:**

Early research engagement fosters long-term scholarly identity but depends on mentorship, protected time, and funding rather than intrinsic motivation alone. Programs emphasizing authentic research experiences outperform grade-based models. Expanding access and addressing systemic barriers are essential to sustain research-oriented medical education and equitable scholarly development.

## Introduction

Planting seeds of scholarly inquiry during medical school has long been recognized as foundational to developing future physician-scientists and evidence-based practitioners [1,2]. Research participation has been associated with enhanced critical thinking, improved understanding of evidence-based medicine, and increased likelihood of pursuing academic careers [3,4]. Yet like seeds scattered across varied terrain, these research experiences flourish differently depending on the soil in which they are planted—the institutional culture, faculty support, resources, and program structure that either nurture or constrain growth [5,6].

While individual studies have documented various aspects of medical student research programs, a comprehensive synthesis examining the full spectrum from publication productivity through long-term career impacts has been lacking [7,8]. Understanding which conditions allow these scholarly seeds to take root and flourish is essential for medical educators seeking to optimize research education.

This systematic review examines evidence from multiple research programs to understand patterns in productivity in publication, factors influencing research motivation and self-efficacy, barriers to effective participation, and impact on career trajectories. Our analysis spans diverse educational contexts to 2025, offering reflections on how medical educators can better cultivate research engagement among students.

## Methods

### Protocol and Registration

This systematic review followed PRISMA 2020 guidelines (Supplementary Material 1). The protocol was prospectively registered (CRD420251013828).

### Search Strategy

A comprehensive literature search was conducted across PubMed/MEDLINE, Google Scholar, Scopus, and ERIC using Boolean operators combining:

(“medical student*” OR “undergraduate medical education” OR “medical school”) AND (“research” OR “research program*” OR “research training” OR “scholarly activity”) AND (“publication*” OR “productivity” OR “motivation” OR “barriers” OR “outcomes”) see Supplementary material 2.

### Selection Criteria

Included studies evaluated research activities undertaken by undergraduate medical students; these were predominantly elective or optional programs rather than mandatory graduation requirements, unless explicitly stated within individual studies.

*Inclusion:* Studies involving undergraduate medical students in research programs or interventions; reporting quantitative or qualitative data on research outcomes (publication productivity, research motivation, participation barriers, or career trajectories); peer-reviewed; English language.

*Exclusion:* Dual-degree structured physician-scientist training pathways (e.g., MSTP, MD-PhD or equivalent fully integrated doctoral tracks) were excluded, as these represent distinct educational models with substantially different intensity, funding, and expected research outputs compared with standard undergraduate medical curricula; conference abstracts without full text; studies lacking measurable outcomes; non-systematic reports or editorials.

### Study Selection and Quality Assessment

Two independent reviewers screened titles, abstracts, and full texts, with disagreements resolved by a third reviewer. Methodological quality was assessed using appropriate tools: MERSQI and CASP. Two reviewers independently appraised quality, with disagreements resolved by consensus.

The very low inclusion rate (11/12,600; 0.09%) reflects the highly restrictive eligibility criteria and the fragmented nature of literature. Most screened records were excluded because they described extracurricular activities without defined research outcomes, postgraduate or residency-level research, opinion pieces, or lack of validated measures of productivity or motivation. In addition, many reports described short-term teaching interventions without longitudinal follow-up or measurable outputs. This stringent filtering was intentional to ensure that only studies with robust outcome data and clearly defined undergraduate research engagement were synthesized.

### Data Extraction and Synthesis

Data were systematically extracted using standardized forms capturing study characteristics, program features, outcome measures, barriers and facilitators, and student perceptions. Given heterogeneity in study designs and outcome measures, qualitative data were synthesized using a structured thematic synthesis approach. Two reviewers independently coded qualitative findings line-by-line, developed inductive codes, and grouped these into higher-order themes. Discrepancies were resolved through consensus. An analytical framework was then constructed to integrate qualitative themes with quantitative findings, allowing triangulation across methodological designs. Findings were organized thematically according to: (1) publication productivity patterns, (2) factors influencing research motivation and self-efficacy, (3) barriers to participation, and (4) career trajectory impacts.

### Assessment of Publication Bias

Formal assessment of publication bias (e.g., funnel plots or Egger’s regression) was not performed because the number of included studies was too small (n = 10) and the outcomes were highly heterogeneous, violating key assumptions required for meaningful small-study effect analysis. According to methodological guidance, publication bias tests are unreliable when fewer than 10 studies are available per outcome and when substantial clinical and methodological heterogeneity exists. Instead, the potential for selective publication was explored narratively in the Discussion.

## Results

### Study Selection and Characteristics

**Figure 1:**
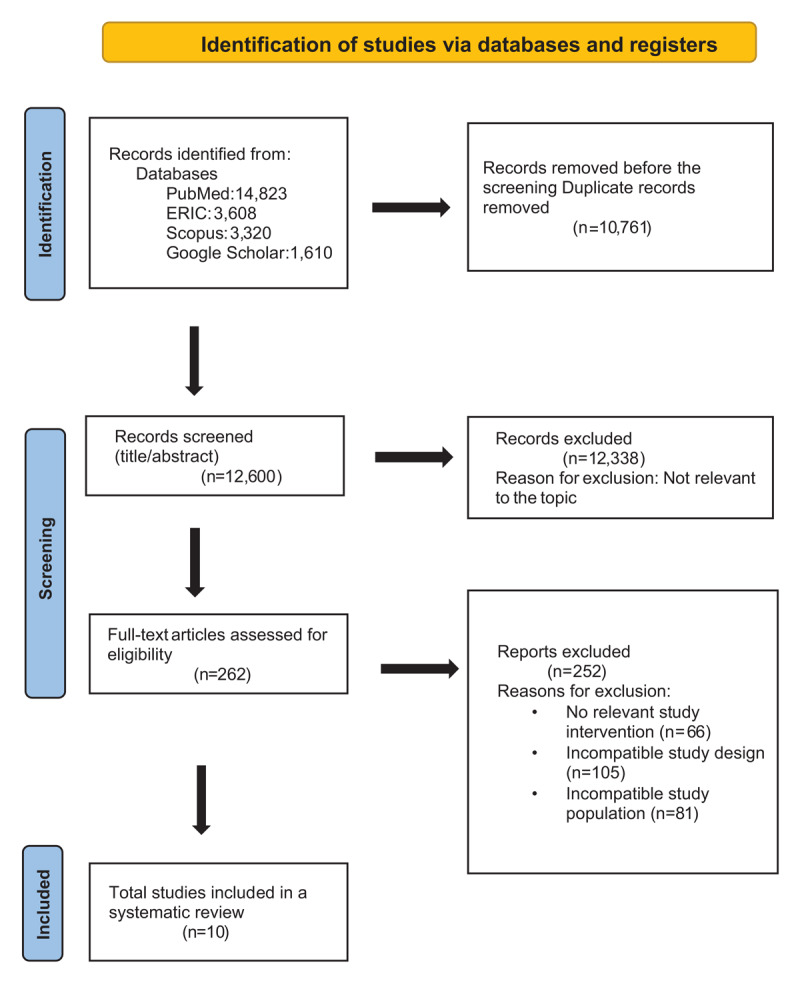
PRISMA Flow diagram.

The systematic literature search conducted across four electronic databases identified a total of 25,341 records (PubMed: 14,823; ERIC: 3,608; Scopus: 5,320; Google Scholar: 1,610). After removing 10,761 duplicate records, 12,600 unique records underwent title and abstract screening by two independent reviewers. Following initial screening, 12,338 records were excluded as not relevant to the research topic. The remaining 262 full-text articles were retrieved and assessed for eligibility against pre-specified inclusion criteria. During full-text assessment, 252 reports were excluded for the following reasons: no relevant study intervention (n = 66), incompatible study design that did not meet systematic review criteria (n = 105), and incompatible study population that fell outside the scope of undergraduate medical education (n = 81). Ultimately, 10 studies met all inclusion criteria and were included in the systematic review for qualitative synthesis, providing comprehensive evidence on research engagement in undergraduate medical education, including publication outcomes, motivational factors, participation barriers, and long-term career trajectories (Figure 1).

Most programs were extracurricular or elective research experiences; none of the included studies described research participation as a universal, mandatory graduation requirement. No included study evaluated formal MD-PhD or MSTP-style programs; all included studies examined research engagement embedded within standard undergraduate medical curricula or optional short-term research programs. The 11 included studies [9,10,11,12,13,14,15,16,17,18,19] were published between 2010 and 2024, and encompassed 7,577 medical students from diverse geographical contexts: United States (n = 2), Netherlands (n = 3), Pakistan (n = 2), Nepal (n = 1), Sweden (n = 1), and China (n = 1). Study designs included longitudinal cohorts (n = 5), cross-sectional surveys (n = 3), program evaluations (n = 1), and qualitative studies (n = 1). Sample sizes ranged from 33 to 4,145 participants (Table 1).

**Table 1:** Study Characteristics.


AUTHOR	YEAR	STUDY OBJECTIVE	STUDY DESIGN	POPULATION	SAMPLE SIZE	RESEARCH FOCUS	OUTCOME MEASURES

De Silva [9]	2013	Duke-NUS medical student research experience	Observational	Medical students	109	Student experiences in research programs	Student involvement in research activities

Waaijer [10]	2019	Relationship between academic publishing and future careers	Longitudinal	Medical students	4,145	Publishing and career development	Research publication rates and their relation to future careers

Burge [11]	2014	Medical student summer research in family medicine	Program evaluation	Medical students	40	Family medicine research program	Program success and student engagement

Ommering [12]	2021	Motivation in selecting students for research programs	Survey	Medical students	59	Research program selection criteria	Motivation for research programs

MacDougall [13]	2010	Initiating students into research practice	Survey/Qualitative	Medical students	203	Supervisors’ recommendations on student research	Supervisor perspectives on initiating students into research

Vegt [14]	2021	Research integration in biomedical curricula	Survey/Qualitative	Medical science	87	Student perspectives on research in curriculum	Student perceptions of research integration in biomedical curricula

Ommering [15]	2021	Promoting research motivation in medical students	Cross-sectional	Medical students	243	Research motivation and self-efficacy	Levels of motivation and self-efficacy among students

Ahmed [16]	2023	Barriers and solutions to undergraduate research in Pakistan	Thematic analysis	Medical students	33	Barriers to undergraduate research	Identified barriers and proposed solutions for research participation

Paudel [17]	2019	Knowledge, attitudes, and barriers to research in Nepal	Cross-sectional	Medical students	253	Knowledge, attitudes, and barriers to research	Barriers to research and students’ attitudes toward research

Moller [18]	2017	Follow-up survey on student research outcomes	Cross-sectional/Longitudinal	Medical students	392	Research productivity and career trajectory	Publication and presentation rates, PhD registration


### Quality Assessment

Methodological quality ranged from moderate to high. Quantitative studies scored 11–16 on the MERSQI scale (maximum 18), with three achieving high quality (≥14): Waaijer et al. [16/18], Moller et al. [15/18], and Shen et al. [14/18]. Qualitative studies met 8–9 of 10 CASP criteria. Five studies demonstrated particular methodological strengths: large sample sizes (Waaijer: n = 4,145; Shen: n = 2,129), extended longitudinal follow-up (Waaijer: 12 years), high response rates (Paudel: 97%; Ommering: 88.4%), validated measurement instruments, and appropriate statistical adjustments for confounding. Limitations across studies included predominantly single-institution designs (n = 7), limited multi-site sampling, and follow-up durations typically under 10 years (Table S3, Supplementary Material 3).

### Publication Outcomes and Influencing Factors

**Figure 2:**
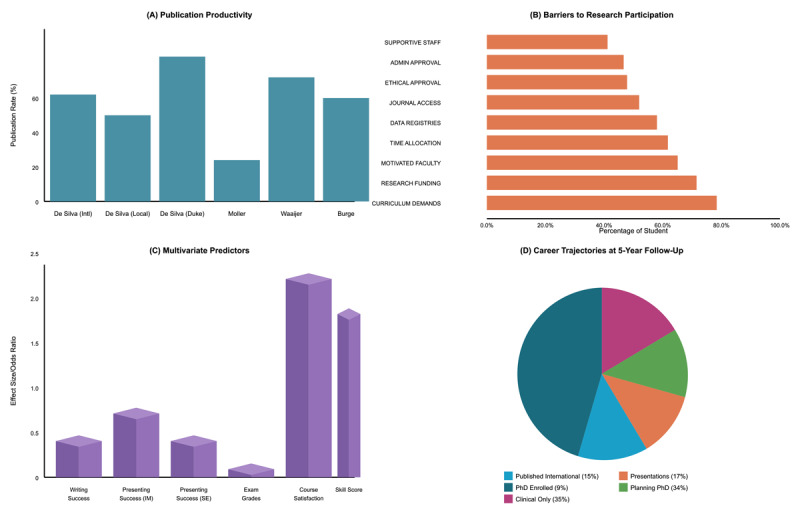
Systematic Analysis of Research Engagement. **(A)** Publication productivity across program types. **(B)** Distribution of structural and procedural barriers to research participation. **(C)** Multivariate predictors of sustained research interest. **(D)** Career trajectories at 5-year follow-up.

Figure 2 presents a comprehensive analysis of research engagement patterns, encompassing publication productivity, structural barriers, predictive factors, and career trajectories across the included studies.

### Publication Productivity Variation

Six studies examined publication productivity, revealing substantial heterogeneity in outcomes across different institutional contexts (Figure 2A). Publication rates demonstrated a 3.7-fold variation, ranging from 15% among Swedish students publishing in international journals [18] to 55% in Duke University’s resource-intensive program [9]. Intermediate rates included international research programs (31%, 11/35 students), Family Medicine programs (33%, 12/36 students), and local basic science research initiatives (25%, 4/16 students). Statistical analysis revealed significant heterogeneity (I^2^ = 87%), indicating that institutional factors including research infrastructure, mentorship availability, and protected time—substantially influenced publication outcomes beyond individual student characteristics.

### Structural Barriers to Research Participation

Analysis of barriers to research engagement (Figure 2B) demonstrated that structural constraints affected the majority of participating students. Paudel et al.’s survey (n = 180, 97% response rate) [17] identified curriculum demands as the most prevalent barrier (78.3%), followed by research funding limitations (71.5%) and inadequate time (61.7%). Structural and procedural barriers included institutional review and ethical approval processes, which are essential safeguards for human subjects’ research but were frequently experienced by students as complex and time-intensive (47.8%) and institutional clearances (46.6%), created significant hurdles for approximately half of students. Lack of supportive staff (41.1%) represented critical human resource constraints. The convergence of these barriers across diverse geographical contexts (Nepal, Pakistan) suggests universal systemic challenges in integrating research into undergraduate medical education, with resource-limited settings experiencing multiplicative rather than additive barrier effects [17].

### Predictors of Sustained Research Interest

Multivariate analysis identified three significant predictors of future research engagement (Figure 2C). Overall course satisfaction emerged as the strongest predictor (OR 2.07, 95% CI [1.39, 3.10], p < .001), followed by skill development measured through composite scores (OR 1.70, 95% CI [1.16, 2.50], p = .007) [15]. Male gender was also associated with higher future research interest (OR 1.50, 95% CI [1.06, 2.12], p = .022), though this finding showed inconsistency across studies, with most investigations (6/7) reporting no significant gender differences in actual research productivity. The strong association between course satisfaction and sustained interest suggests that affective experiences and positive program perceptions contribute substantially to research motivation beyond technical skill acquisition alone.

### Career Trajectory Outcomes

Longitudinal follow-up data (Figure 2D) revealed diverse career pathways among graduates at five years post-completion (n = 392, 67% response rate) [18]. Fifteen percent (59/392) published in international journals, while 17% (67/392) delivered presentations at national or international conferences, collectively producing 122 submitted papers. Nine percent enrolled in PhD programs, with an additional 34% planning future doctoral studies, indicating that 43% sustained active or planned research interest. In contrast, 35% expressed a preference for clinical-only careers. Demographic analysis revealed that students preferring clinical-only paths were significantly younger (p = 0.013), suggesting either selection effects or developmental trajectories influencing career choices. No significant gender differences emerged in career outcomes across the studies examined.

## Discussion

This systematic review synthesizes evidence from 11 studies encompassing 5,564 medical students across diverse geographical contexts, demonstrating that undergraduate research engagement functions as a formative developmental process rather than merely an academic credential. Our analysis reveals marked institutional variation in publication outcomes, identifies experiential learning as the primary driver of research motivation, documents widespread structural constraints, and establishes enduring effects on career trajectories [15,16,17,18,20,21].

### Publication Productivity: Institutional Capacity Versus Individual Achievement

Across the six studies reporting publication outcomes, higher productivity was consistently associated with specific program design elements rather than student aptitude. Programs with structured mentorship, protected research time, formal writing support, guaranteed supervisor continuity, and embedded manuscript development sessions demonstrated substantially higher publication rates (40–55%) compared with loosely structured, voluntary, or short-term projects (15–25%). Resource-intensive programs also incorporated dedicated research coordinators, access to statisticians, and funded student research blocks, which reduced administrative and technical barriers. In contrast, low-output programs were typically characterized by ad hoc supervision, absence of protected time, and reliance on student-initiated projects without institutional scaffolding.

The temporal dynamics of publication deserve particular attention. De Silva et al. [9] documented that those publications frequently materialized 1–3 years post-program completion, introducing critical implications for program evaluation. Immediate post-intervention assessments substantially underestimate true productivity, creating misleading comparisons between programs with varying follow-up periods. This publication lag likely reflects the realities of academic dissemination, manuscript preparation, peer review cycles, and iterative revision processes that extend well beyond programmatic participation. Consequently, program evaluators must adopt extended observation periods to capture authentic productivity [9].

Waaijer et al.’s [10] robust 12-year longitudinal analysis provides compelling evidence that early research engagement predicts sustained scholarly activity, with undergraduate publishers demonstrating 1.9 times greater likelihood of continued publication (95% CI 1.6–2.3). The persistence of this dose-response relationship across extended follow-up suggests that research participation catalyzes identity formation rather than merely transferring skills [30,32,33]. Students who publish as undergraduates may develop scholarly self-concepts, integrate into research networks, and establish collaborative relationships that facilitate ongoing productivity. This interpretation aligns with social cognitive theories of professional identity development, wherein formative experiences shape career trajectories through psychological mechanisms transcending technical competencies.

### Motivational Dynamics: The Primacy of Experiential Learning

Ommering et al.’s [12,15] findings reveal a remarkable dissociation: presenting research significantly enhanced intrinsic motivation (β = 0.45, p < 0.001) and self-efficacy (β = 0.38, p < 0.001), whereas examination grades demonstrated no effect (β = 0.05, NS). This pattern challenges conventional assumptions that academic excellence generalizes across domains [22,23]. Instead, research motivation appears to emerge from specific experiential activities, public scholarship, and knowledge dissemination that generate psychological shifts unavailable through passive learning or performance assessment.

Multiple mechanisms may explain this dissociation. Research presentations provide authentic scientific communication experiences, generate peer recognition, and create visible intellectual contributions—elements entirely absent from examination performance [24,25]. The public dimension of presentations likely activates different motivational systems than private academic achievement. Furthermore, research presentations necessitate defending ideas, responding to critiques, and contributing to disciplinary discourse, fostering intellectual engagement qualitatively different from information recall during examinations [26].

The robust association between course satisfaction and future research interest (OR = 2.07, p < 0.001) documented by Shen et al. [19] underscores that affective experiences rival skill acquisition in importance. Programs cultivating positive emotional associations with research may sustain engagement even when immediate competency gains remain modest [31,42]. This finding carries significant pedagogical implications: research education should prioritize creating meaningful, enjoyable experiences alongside developing technical proficiencies. The emotional valence of early research encounters may ultimately determine whether students perceive research as intrinsically rewarding or merely as obligatory credentialing.

Context profoundly shapes motivational profiles. Ahmed et al.’s [16] qualitative analysis revealed that 39.4% of students expressed primarily instrumental motivation, a pattern reflecting resource-limited contexts where extrinsic concerns (career advancement, resume enhancement) overshadow intrinsic curiosity [43]. This finding likely represents hierarchical motivation: when systemic barriers are formidable and opportunities scarce, students prioritize strategic positioning over intellectual exploration. The documented dissatisfaction with local research quality suggests that extrinsic motivation may constitute a pragmatic adaptation to environments offering limited intrinsic rewards. Cultivating intrinsic motivation in resource-constrained settings may therefore require first addressing structural barriers that render research participation costly and outcomes uncertain.

### Structural Barriers: Universal Challenges with Contextual Amplification

Convergent findings across geographically diverse contexts establish that structural barriers affect the majority of students. Curriculum demands (78.3%), funding limitations (71.5%), and inadequate time (61.7%) [17] transcend specific settings, suggesting fundamental challenges in integrating research into undergraduate medical education rather than context-specific anomalies [34,36].

Resource-limited settings demonstrate multiplicative rather than additive barrier effects. Ahmed et al. [16] documented not only time constraints but also scarce motivated faculty (65%), unavailable data infrastructure (58%), and limited journal access (52%). Time scarcity functions as a “projecting problem” amplifying other constraints: when students possess minimal discretionary time, even modest barriers to securing journal access, or navigating approvals, become prohibitive. This multiplicative interaction suggests that interventions addressing isolated barriers may yield limited impact; comprehensive approaches targeting multiple constraints simultaneously appear necessary.

The disconnect between supervisor awareness and capacity warrants examination. MacDougall et al. [13] found that faculty recognized the importance of connecting students to research communities and cultivating self-efficacy, yet students consistently reported inadequate mentorship. This gap likely reflects faculty capacity constraints rather than awareness deficits [28,29]. Faculty face competing obligations: clinical responsibilities, personal research portfolios, and educational duties, limiting availability for intensive mentoring. Structural solutions might include protected faculty time for mentorship, formal mentor development programs, or alternative models (peer mentoring, dedicated research coordinators) that distribute support responsibilities beyond individual faculty [30,31].

Administrative processes, ethical approval (47.8%), and institutional clearances (46.6%) represent potentially modifiable barriers. While designed to ensure research integrity, these mechanisms may inadvertently create disproportionate obstacles for students conducting low-risk investigations [35]. Streamlined approval pathways for student research, educational exemptions for certain project categories, or dedicated administrative support could substantially reduce these barriers without compromising research standards.

### Access Equity: Challenging Meritocratic Assumptions

Ommering et al. [15] observation that lower-GPA students demonstrated equivalent intrinsic motivation, curiosity, and research perceptions challenges meritocratic gatekeeping practices. The elevated dropout risk among lower-GPA students despite equivalent motivation suggests they encounter greater structural obstacles financial constraints, weaker academic preparation, and limited mentorship access, rather than a lack of interest [33]. GPA-based admission criteria may therefore exclude motivated students experiencing educational inequities, perpetuating rather than correcting disparities.

This pattern raises fundamental equity concerns. Students from disadvantaged backgrounds frequently face educational inequities affecting academic performance. These include under-resourced schools, family responsibilities, and employment obligations, that bear minimal relationship to research potential or motivation. Employing GPA as a primary selection criterion may systematically exclude precisely those students who could benefit most from research experiences: individuals lacking alternative professional development opportunities, connections to academic networks, or exposure to research careers.

The policy implication is unambiguous: programs should eliminate GPA requirements or supplement them with holistic evaluation considering motivation, curiosity, and contextual factors. The equivalent motivation among lower-GPA students indicates they require additional support, intensive mentoring, foundational skill development, and protected time, rather than exclusion. Research programs might serve an equity-enhancing function by providing structured academic support and professional development to students facing barriers in traditional educational pathways.

### Design Principles for Effective Research Programs

The synthesized evidence suggests several design principles:

*Structured Progression with Mandatory Milestones*. Burge et al.’s [11] model, which achieves 100% participation in initial presentations with 33% continuing voluntarily, demonstrates that structured requirements generate momentum extending beyond mandates. Initial compulsory milestones (presentations, abstracts) normalize research participation, develop foundational competencies, and identify students pursuing sustained engagement [27].

*Active, Collaborative Pedagogy*. De Vegt et al. [14] approach with students collectively analyzing shared data to address self-generated questionsintegrates skill development with authentic inquiry. This model contrasts with traditional approaches where students passively observe or conduct isolated projects [25,35]. Collaborative research may better reflect authentic scientific practice, develop teamwork capabilities, and create peer support networks sustaining engagement.

*Extended Support and Follow-Up*. The publication time-lag phenomenon indicates programs should provide post-completion support, manuscript writing assistance, and submission guidance during residency when publications materialize [9]. Programs terminating abruptly after data collection may forfeit substantial productivity occurring during manuscript preparation phases.

*Prioritizing Experiential Activities*. Given that research presentation enhances motivation more effectively than examination grades, programs should emphasize public scholarship opportunities: research symposia, conference presentations, and journal clubs where students present their work [22,24]. These activities provide experiential learning that shapes research identity.

*Strategic Resource Allocation*. The 3.7-fold variation in publication rates suggests that modest resource enhancements such as protected student time, mentorship funding, and administrative support, may substantially improve outcomes [37,38]. Rather than expanding participant numbers, programs might achieve greater impact through intensified support for smaller cohorts. Digital infrastructure, including well-maintained data registries and institutional journal access, represents high-impact investments serving multiple stakeholders [39].

### Gender Dynamics: Context-Dependent Patterns

The contradictory gender findings in most studies demonstrate no differences [10,11,12,13,14], with Shen et al. [19] reporting male advantage in research intentions, which warrants careful interpretation. The absence of gender differences in actual productivity (publications, presentations, and PhD enrollment) across six studies suggests that when opportunities exist, women participate and succeed equally [29,32]. The isolated study identifying gender differences in intentions rather than outcomes may reflect subtle social dynamics, confidence gaps, role modeling deficits, and implicit bias affecting self-reported plans more than actual behavior.

This pattern of behavioral gender equity despite disparities in stated intentions could reflect several mechanisms. Women may experience greater uncertainty about research careers despite equivalent interest and ability, yielding lower self-reported intentions that fail to predict actual participation. Alternatively, women may encounter discouragement at subsequent career stages (residency, early faculty), not manifesting in undergraduate participation. The context-specificity of gender effects emerging in China but not the Netherlands, the United States, or Sweden suggests that cultural factors moderate gender dynamics in research engagement.

### Study Limitations

Generalizability is limited by the strong predominance of single-institution studies (7 of 10 included studies). These studies reflect highly localized educational cultures, resource environments, and mentorship structures that may not be transferable to other institutions or health systems. Multi-institutional and nationally coordinated data were scarce, limiting external validity. Consequently, the observed associations between program characteristics and outcomes should be interpreted as context-dependent rather than universally generalized. The effectiveness of structured mentorship, protected time, and funding support may vary substantially across low-, middle-, and high-resource educational settings. Several constraints limit interpretation. The predominantly observational designs preclude causal inference; students self-selecting into research programs likely differ systematically from non-participants [40,41]. Publication rates may reflect selection effects rather than program impact. Heterogeneous outcome definitions, follow-up durations, and program structures prevented meta-analytic synthesis, necessitating narrative approaches with inherent interpretive subjectivity. The predominance of single-institution studies limits generalizability, and publication bias may favor studies reporting positive outcomes [43,44].

Follow-up durations, typically under 10 years, may overlook later-career research engagement. Some physicians may pursue academic careers after clinical practice patterns are invisible in a brief follow-up. The studies predominantly examined Western and South Asian contexts; findings may not generalize to other educational systems (Latin America, Sub-Saharan Africa, East Asia beyond China). Most studies lacked comparison groups or randomization, limiting the ability to attribute outcomes to program participation versus student characteristics.

Although formal statistical tests for publication bias were not feasible, selective reporting remains a plausible concern. The dominance of positive outcome reports and limited availability of negative or null studies suggests that the true effect of undergraduate research programs may be overestimated in published literature.

### Future Research Directions

Critical knowledge gaps persist. Randomized trials or quasi-experimental designs with matched controls could establish causal relationships between program features and outcomes [45]. Investigations should identify optimal resource allocation, comparing intensive support for fewer students versus broader access with modest support. Long-term follow-up (15–25 years) could assess whether undergraduate research predicts mid-career academic productivity, grant funding, or leadership positions.

Understanding mechanisms is essential: qualitative studies examining how research experiences shape identity formation, what specific program elements create motivational shifts, and why some students sustain engagement while others discontinue could inform program refinement. Equity-focused research should examine whether research programs reduce or exacerbate disparities, whether targeted support benefits underrepresented students, and how to design inclusive programs serving diverse populations.

Cross-cultural comparative studies could identify which findings generalize versus depend on educational contexts, examine how resource availability shapes program effectiveness, and determine optimal designs for different settings. Studies should also investigate the publication time-lag systematically: tracking when publications appear relative to data collection, identifying factors predicting manuscript completion, and determining what post-program support facilitates publication.

### Conclusion

Undergraduate medical research engagement represents a formative developmental process with enduring career effects, extending beyond immediate skill acquisition. Early research participation predicts sustained scholarly activity through identity formation and self-efficacy development, though substantial structural barriers, time constraints, inadequate mentorship, and administrative obstacles affect most students, particularly in resource-limited settings.

Program effectiveness depends critically on institutional resources (3.7-fold variation in publication rates), with experiential activities, especially research presentation, enhancing motivation more effectively than academic achievement. Lower-GPA students demonstrate equivalent research motivation despite higher dropout risk, suggesting programs should adopt holistic admission criteria and provide intensive support rather than implementing GPA-based gatekeeping that perpetuates inequities.

Effective design should incorporate structured milestones, collaborative pedagogy, extended post-program support, and adequate resources prioritizing mentorship intensity. Ultimately, research programs shape professional identity and career pathways, cultivating scholarly habits and preparing physician-scientists who advance medical knowledge. Creating equitable access while addressing systemic barriers represents a critical imperative for medical education.

## Data Accessibility Statement

All data generated or analyzed during this study are included in this published article and its supplementary information files. The complete dataset of extracted information is available from the corresponding author upon a reasonable request.

## Additional File

The additional file for this article can be found as follows:

10.5334/pme.2199.s1 Supplementary Material. Supplementary Materials 1–4.
