# Supplementary material for: Seeds of Scholarship: A Systematic Review of Research Engagement in Undergraduate Medical Education
Source: Perspect Med Educ. 2026 Jan 21;15(1):16–27. doi: 10.5334/pme.2199 (PMC12829458; doi:10.5334/pme.2199)
Supplement: Supplementary Material. — Supplementary Materials 1–4. [file pme-15-1-2199-s1.pdf]

Supplementary Material

Supplementary Material 1: PRISMA Checklist

Supplementary Material 2: Search Strategy for All Databases

Supplementary Material 3: Quality Assessment

Supplementary Material 4: Predictors of Research Interest

Supplementary Material 1

(PRISMA Checklist)

Table S1: PRISMA Checklist

Topic	No.	Item	The location where item is reported
**TITLE**			
**Title**	1	Identify the report as a systematic review.	Page1
**ABSTRACT**			
**Abstract**	2	See the PRISMA 2020 for the Abstracts checklist	Page 2
**INTRODUCTION**			
**Rationale**	3	Describe the rationale for the review in the context of existing knowledge.	Page 4
**Objectives**	4	Provide an explicit statement of the objective(s) or question(s) the review addresses.	Page 4
**METHODS**			
**Eligibility criteria**	5	Specify the inclusion and exclusion criteria for the review and how studies were grouped for the syntheses.	Page 45
**Information sources**	6	Specify all databases, registers, websites, organizations, reference lists, and other sources searched or consulted to identify studies. Specify the date when each source was last searched or consulted.	Page 5
**Search strategy**	7	Present the full search strategies for all databases, registers, and websites, including any filters and limits used.	Page 4-5 and Supplementary material 2

**Selection process**	8	Specify the methods used to decide whether a study met the inclusion criteria of the review, including how many reviewers screened each record and each report retrieved, whether they worked independently, and if applicable, details of automation tools used in the process.	Page 5
**Data collection process**	9	Specify the methods used to collect data from reports, including how many reviewers collected data from each report, whether they worked independently, any processes for obtaining or confirming data from study investigators, and if applicable, details of automation tools used in the process.	Page 5
**Data items**	10a	List and define all outcomes for which data were sought. Specify whether all results that were compatible with each outcome domain in each study were sought (e.g. for all measures, time points, analyses), and if not, the methods used to decide which results to collect.	Page 5
	10b	List and define all other variables for which data were sought (e.g. participant and intervention characteristics, funding sources). Describe any assumptions made about any missing or unclear information.	Page 5
**Study risk of bias assessment**	11	Specify the methods used to assess risk of bias in the included studies, including details of the tool(s) used, how many reviewers assessed each study and whether they worked independently, and if applicable, details of automation tools used in the process.	Page 5
**Effect measures**	12	Specify for each outcome the effect measure(s) (e.g. risk ratio, mean difference) used in the synthesis or presentation of results.	N/A
**Synthesis methods**	13a	Describe the processes used to decide which studies were eligible for each synthesis (e.g. tabulating the study intervention characteristics and comparing against the planned groups for each synthesis (item 5)).	Page 5
	13b	Describe any methods required to prepare the data for presentation or synthesis, such as handling of missing summary statistics, or data conversions.	Page 5
	13c	Describe any methods used to tabulate or visually display results of individual studies and syntheses.	Page 5
	13d	Describe any methods used to synthesize results and provide a rationale for the choice(s). If meta-analysis was performed, describe the model(s), method(s) to identify the presence and extent of	Page 5

		statistical heterogeneity, and software package(s) used.	
	13e	Describe any methods used to explore possible causes of heterogeneity among study results (e.g. subgroup analysis, meta-regression).	N/A
	13f	Describe any sensitivity analyses conducted to assess robustness of the synthesized results.	N/A
**Reporting bias assessment**	14	Describe any methods used to assess risk of bias due to missing results in a synthesis (arising from reporting biases).	N/A
**Certainty assessment**	15	Describe any methods used to assess certainty (or confidence) in the body of evidence for an outcome.	N/A
**RESULTS**			
**Study selection**	16a	Describe the results of the search and selection process, from the number of records identified in the search to the number of studies included in the review, ideally using a flow diagram.	Page 6 and Figure 1
	16b	Cite studies that might appear to meet the inclusion criteria, but which were excluded, and explain why they were excluded.	Page 6
**Study characteristics**	17	Cite each included study and present its characteristics.	Page 6 and Table 1
**Risk of bias in studies**	18	Present assessments of risk of bias for each included study.	Page 9 and Supplementary Material 3
**Results of individual studies**	19	For all outcomes, present, for each study: (a) summary statistics for each group (where appropriate) and (b) an effect estimates and its precision (e.g., confidence/credible interval), ideally using structured tables or plots.	Page 11-16 and Supplementary Material 4
**Results of syntheses**	20a	For each synthesis, briefly summarise the characteristics and risk of bias among contributing studies.	Supplementary Material 3
	20b	Present results of all statistical syntheses conducted. If meta-analysis was done, present for each the summary estimate and its precision (e.g. confidence/credible interval) and measures of statistical heterogeneity. If comparing groups, describe the direction of the effect.	N/A
	20c	Present results of all investigations of possible causes of heterogeneity among study results.	N/A

	20d	Present results of all sensitivity analyses conducted to assess the robustness of the synthesized results.	N/A
**Reporting biases**	21	Present assessments of risk of bias due to missing results (arising from reporting biases) for each synthesis assessed.	N/A
**Certainty of evidence**	22	Present assessments of certainty (or confidence) in the body of evidence for each outcome assessed.	N/A
**DISCUSSION**			
**Discussion**	23a	Provide a general interpretation of the results in the context of other evidence.	Page 16
	23b	Discuss any limitations of the evidence included in the review.	Page 23
	23c	Discuss any limitations of the review processes used.	Page 23
	23d	Discuss the implications of the results for practice, policy, and future research.	Page 24
**OTHER INFORMATION**			
**Registration and protocol**	24a	Provide registration information for the review, including the register name and registration number, or state that the review was not registered.	Page 4
	24b	Indicate where the review protocol can be accessed, or state that a protocol was not prepared.	Page 4
	24c	Describe and explain any amendments to information provided at registration or in the protocol.	Page 4
**Support**	25	Describe sources of financial or non-financial support for the review, and the role of the funders or sponsors in the review.	Page 25
**Competing interests**	26	Declare any competing interests of review authors.	Page 25
**Availability of data, code, and other materials**	27	Report which of the following are publicly available and where they can be found template data collection forms; data extracted from included studies; data used for all analyses; analytic code; any other materials used in the review.	Page 25

From: Page MJ, McKenzie JE, Bossuyt PM, Boutron I, Hoffmann TC, Mulrow CD, et al. The PRISMA 2020 statement: an updated guideline for reporting systematic reviews. MetaArXiv. 2020, September 14. DOI: 10.31222/osf.io/v7gm2. For more information, visit: www.prisma-statement.org

Supplementary Material 2

(Search Strategy for All Databases)

Table S2: Search Strategy

Search strategy	Results
**PubMed**	
Search: undergraduate medical education Filters: from 2000 - 2025	34,043
Search: undergraduate medical education AND (research training) Filters: from 2000 - 2025	14,823
Search: undergraduate medical education AND (publication productivity) Filters: from 2000 - 2025	496
Search: undergraduate medical education AND (research motivation) productivity) Filters: from 2000 - 2025	1,184
Search: undergraduate medical education AND (barriers to research) productivity) Filters: from 2000 - 2025	885
ERIC	
Search: undergraduate medical education	1,506
Search: undergraduate medical education AND “research training”	3,608
Search: undergraduate medical education AND “publication productivity”	91
Search: undergraduate medical education AND “research motivation”	502
Search: undergraduate medical education AND “barriers to research’	125
**Scopus**	
Search documents: undergraduate medical education Filters: Range 2000 - 2025	35,869
Search documents: undergraduate medical education AND research training Filters: Range 2000 - 2025	3,320
Search documents: undergraduate medical education AND publication productivity Filters: Range 2000 - 2025	43
Search documents: undergraduate medical education AND research motivation Filters: Range 2000 - 2025	589
Search documents: undergraduate medical education AND barriers to research Filters: Range 2000 - 2025	597
**Google Scholar**	
Search: “undergraduate medical education” Custom range 2000-2025	39,000
Search: “undergraduate medical education” AND “research training” Custom range 2000-2025	1,610
Search: “undergraduate medical education” AND “publication productivity” Custom range 2000-2025	52
Search: “undergraduate medical education” AND “research motivation” Custom range 2000-2025	68
Search: “undergraduate medical education” AND “barriers to research” Custom range 2000-2025	191

Supplementary Material 3

(Quality Assessment)

Table S3: Risk of Bias Score for Quantitative Impact Studies

Study	Year	Study Design	Assessment Tool	Quality Rating	Key Strengths	Key Limitations
De Silva	2013	Quantitative (Program evaluation)	MERSQI	14/18	Clear objectives: publication outcomes tracked	Small sample; short-term follow-up
Burge	2014	Mixed methods	MERSQI + CASP	13/18	Consistent productivity data: student perception included	Limited generalizability
MacDougall	2010	Qualitative	CASP	High	Rich thematic analysis; supervisor perspectives	Limited reflexivity
Moller	2017	Quantitative longitudinal	MERSQI	16/18	Large sample; long-term follow-up	Voluntary reporting bias
Paudel	2019	Quantitative (survey)	MERSQI	12/18	High response rate	Self-report; limited statistical analysis
Waaijer	2019	Quantitative (national cohort)	MERSQI	15/18	Robust data linkage; objective outcomes	Limited qualitative insight
Ommering	2021a	Quantitative (cohort)	MERSQI	15/18	GPA-adjusted analysis; validated measures	Single institution
Ommering	2021b	Quantitative (motivational study)	MERSQI	14/18	Focused motivation analysis	Cross-sectional design

Vegt	2021	Mixed methods	MERSQI + CASP	13/18	Student perception of active learning	Limited sample representativeness
Ahmed	2023	Qualitative (thematic)	CASP	Moderate	In-depth exploration of barriers	Regional limitation: small sample

CASP: Critical Appraisal Skills Programme, Medical Education Research Study Quality Instrument

Supplementary Material 4

(Predictors of Research Interest)

Table S4: Predictors of Research Interest in Medical Students

Predictor	Outcome	Statistics	P-value	Note
Overall course satisfaction	Future research interest	OR 2.07 95%CI [1.39, 3.10]	< .001	Strong predictor
Skill development (composite scores)	Future research interest	OR 1.07 95%CI [1.16, 2.50]	.007	-
Male gender	Future research interest	OR 1.50 95%CI [1.06, 2.12]	.022	Motivational only

OR = Odds Ratio; CI = Confidence Interval; Statistical *significance set at p < .05*
